# Mechanical and Water-Resistant Properties of Eco-Friendly Chitosan Membrane Reinforced with Cellulose Nanocrystals

**DOI:** 10.3390/polym11010166

**Published:** 2019-01-18

**Authors:** Haiquan Mao, Chun Wei, Yongyang Gong, Shiqi Wang, Wenwen Ding

**Affiliations:** 1 College of Materials Science and Engineering, Guilin University of Technology, Guilin 541004, China; lovedust@163.com (H.M.); wangshiqixhh@gmail.com (S.W.); ding291636041@163.com (W.D.); 2 Key Laboratory of New Processing Technology for Nonferrous Metals and Materials, Ministry of Education, Guilin University of Technology, Guilin 541004, China

**Keywords:** cellulose nanocrystals, chitosan, membrane, water resistance, mechanical property

## Abstract

Environmentally benign and biodegradable chitosan (CS) membranes have disadvantages such as low mechanical strength, high brittleness, poor heat resistance and poor water resistance, which limit their applications. In this paper, home-made cellulose nanocrystals (CNC) were added to CS to prepare CNC/CS composite membranes through mechanical mixing and solution casting approaches. The effects of CNC dispersion patterns and CNC contents on the properties of composite membranes were studied. The analysis of the surface and cross-section morphology of the membranes showed that the dispersion performance of the composite membrane was better in the case that CNC was dissolved in an acetic acid solution and then mixed with chitosan by a homogenizer (Method 2). CNC had a great length-diameter ratio and CNC intensely interacted with CS. The mechanical properties of the composite membrane prepared with Method 2 were better. With a CNC content of 3%, the tensile strength of the composite membrane reached 43.0 MPa, 13.2% higher than that of the CNC-free membrane. The elongation at break was 41.6%, 56.4% higher than that of the CNC-free membrane. Thermogravimetric, contact angle and swelling analysis results showed that the addition of CNC could improve the heat and water resistance of the chitosan membrane.

## 1. Introduction

In the 66 years between 1950 and 2015, the world produced 8.3 billion tons of plastic, but 6.3 billion tons of which went to waste. Only 9% was recycled, 12% was burned, and 79% was buried in soil or dispersed in nature [1]. On the other hand, 99% of the plastics were produced from unsustainable petroleum [2]. In spite of excellent properties, these are not recyclable and difficult to biodegrade. As a result, limited petroleum resources are consumed, the environment is polluted, and human health and the ecological environment are endangered.

Biopolymers are considered to be the most promising alternatives to petroleum-based polymers, because they can greatly reduce the dependence on petroleum and reduce environmental pollution. In recent years, the demands for bio-derivative and biodegradable packaging materials have been significantly increased [3]. Chitosan is abundant and widespread in the world (its amount is only smaller than that of cellulose in nature). From a chemical point of view, chitosan is a renewable and biodegradable cationic polymer. Besides, chitosan is the only polysaccharide containing a great many basic groups (–NH_2_) in nature. Many studies have disclosed that chitosan possesses many unique physical, chemical and biological properties, such as safety, non-toxicity, good antimicrobial performance, biocompatibility and excellent membrane-forming performance [4,5]. The molecular weight of chitosan ranges from tens of thousands to millions, and Chitosan is insoluble in water but soluble in acetic acid solution. Because of the great number of amino and hydroxyl groups in chitosan, under specific conditions, chemical reactions such as alkalization, acylation, esterification, etherification, alkylation, oxidation, hydrolysis, cross-linking and grafting copolymerization [6] can occur to form a variety of chitosan derivatives with different physical and chemical properties and biological functions. These derivatives can be applied in the fields of biomedicine [7,8,9], environmental protection [10,11], food packaging [12], functional materials [13,14], membrane technology [15,16,17,18] and so on. For example: Zhijiang, C. et al. [19] prepared a chitosan-based nanofiltration membrane by electrospinning technology, which can effectively remove the dye in wastewater [20]. The cellulose and chitosan composite membrane prepared by Urbina, L. [21] and Lam, B. [22] et al. can effectively remove heavy metal ions in water. In addition, a large number of studies have shown that the chitosan composite membrane has good anti-adhesion [23], coagulation [24,25] and antibacterial [26,27] effects. However, a membrane consisting of chitosan alone has disadvantages such as low mechanical strength, high brittleness, poor heat resistance and poor water resistance, which limit the applications of chitosan [19,28,29,30].

**Figure 1:**
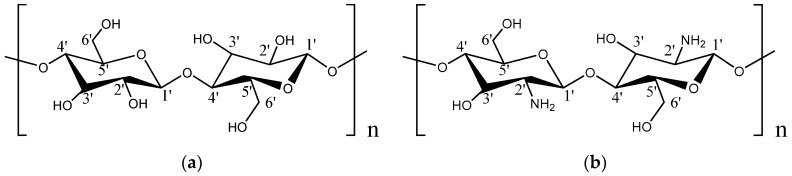
(**a**) Cellulose molecule, (**b**) Chitosan molecule.

As a renewable and biodegradable natural polysaccharide polymer, cellulose is the most widespread and abundant polysaccharide in nature. Cellulose and chitosan have similar molecular structures, as shown in Figure 1 (except that the C2 site in cellulose is –OH while that in chitosan is –NH_2_, the remaining parts are the same). Nano-cellulose can be refined into smaller nano-crystals with greater crystallinity through the removal of amorphous parts in cellulose by using acidic or enzymatic catalysis. The cellulose nanocrystals have many excellent properties, such as non-toxicity, good biocompatibility, high crystallinity, high transparency, high hydrophilicity, tensile strength as high as 7500 MPa, and Young’s modulus as high as 140 GPa [31]. Additionally, cellulose molecules contain a large number of hydroxyl groups, which are subject to oxidation, esterification, etherification and other reactions. Thanks to these hydroxyl groups, hydrogen bonds can be formed between the molecules, and cellulose is easy to graft and copolymerize with other substances. These properties ensure the wide applications of cellulose in paper industry [32], food packaging [33], biomedicine [34], adsorbent [35,36], new energy [37,38,39], etc. Nano-cellulose with large aspect ratio as a dispersion phase can enhance natural or synthetic polymers and can improve the mechanical properties [30,40,41], water resistance [42,43], heat resistance [44], and so on. Namely, composite materials with excellent properties can be derived.

**Figure 2:**
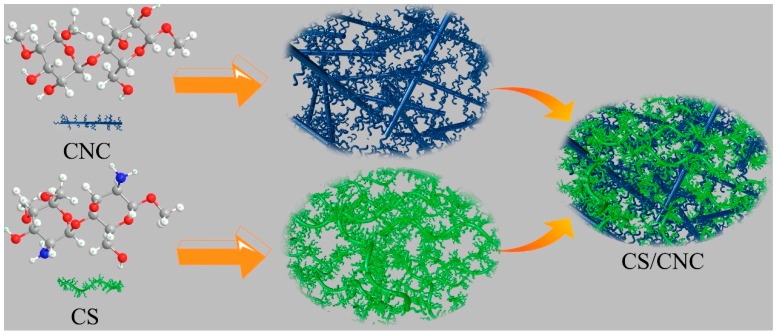
The process of forming polyelectrolyte complex (PEC) between cellulose nanocrystals (CNC) and chitosan (CS) molecules.

Chitosan is a cationic polymer and nano-cellulose is an anionic polymer. Nano-composites can be prepared by blending chitosan and nano-cellulose, both of which interact with each other to spontaneously form a polyelectrolyte complex (PEC) in an aqueous solution, as shown in Figure 2. Strong electrostatic association is the main interaction type, and hydrogen bond, hydrophobic interaction, dipole interaction, van der Waals force and other interaction types also contribute [7]. The formation and properties of PEC are affected by many parameters, including skeleton structure, molecular weight, degree of substitution of ionic groups, charge density, mixing procedure and rate, ionic strength, pH and solution temperature. Many studies have shown that the composite of cellulose and chitosan can achieve excellent performance of the composite membrane. For example, Huang, X. et al. [45] used surface-modified microcrystalline cellulose (MCC) to enhance the chitosan film. When the MCC content was 7 wt %, the tensile strength reached 59.1MPa, which was 97.6% higher than that of the chitosan film, but its elongation at break is only 11.5%. Chi, K. et al. [30] prepared a polyelectrolyte composite (PPC) which was compounded by chitosan (CS), cellulose nanocrystals (CNC), and carboxymethyl cellulose (CMC). The tensile strength of the PPC film can reach 60.6 MPa, which is 40% higher than that of the CS film, and 52% higher than that of the CMC film, but the elongation at break is only 1.7%–2.4%. Although they all increase the strength of the composite film, the elongation at break can still be very low, and this does not really solve the problem of chitosan brittleness. 

In this paper, CNC prepared by sulfuric acid hydrolysis was added to CS, and the composite film was prepared by solution casting process. The effects of different dispersion patterns and CNC content on the structure and mechanical properties of CNC/CS composite membranes were studied. The results show that CNC can improve the strength, elongation at break, thermal properties and water resistance of chitosan membrane, which are informative for new environmentally benign membrane materials.

## 2. Materials and Methods

### 2.1. Materials

Chitosan (deacetylation degree was >95%; viscosity was 100–200 mpa·s) and acetic acid (99.5%, AR) were purchased from Aladdin Chemicals Co., Ltd. (Shanghai, China). CNC and deionized water were homemade.

### 2.2. Methods

#### Preparation of CNC/CS Composite Membrane

**Figure 3:**
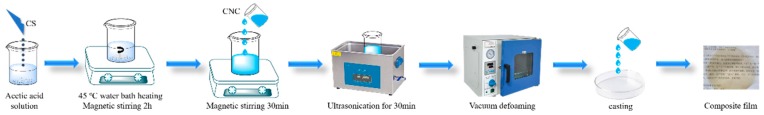
Preparation procedure of composite membranes with Method 1.

Method 1: As shown in Figure 3, a 2% chitosan (CS) solution was prepared. In detail, 6 g of acetic acid was added into 288 g of deionized water. Then, 6 g of chitosan was added. The mixture was stirred and heated with a water bath at 45 °C for 2 h, until the chitosan was completely dissolved. Different volumes of the 0.7% light blue clear CNC solution were separately added to 40 mL of the 2% chitosan solution, and the corresponding CNC mass fractions in these composite membranes were 0%, 1%, 2%, 3%, 4% and 5%. These solutions were stirred and heated at 45 °C for 0.5 h, and then sonicated for 0.5 h, for the homogeneous mixture of CNC and CS. These solutions treated were maintained for 4 h, and then were defoamed in a vacuum drying oven for 30 min. Then, the solutions were poured into petri dishes to cast into membranes, which were placed into the drying oven at 50 °C. After 3 days, transparent composite membranes were obtained.

**Figure 4:**
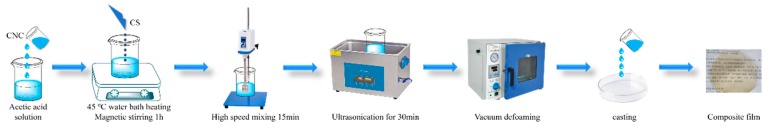
Preparation procedure of composite membranes with Method 2.

Method 2: As shown in Figure 4, different volumes of the 0.7% CNC solution were separately added to 38.4 mL of deionized water, and the corresponding CNC mass fractions in these composite membranes were 0%, 1%, 2%, 3%, 4% and 5% and then mixed with 0.8 g of acetic acid. 0.8 g of chitosan subsequently were added to the solutions with slow stirring. These mixtures were stirred and heated at 45 °C for 1 h to fully dissolve the chitosan. The mixtures were stirred at a high rate with a homogenizer for 15 min, and were then sonicated for 30 min. After being maintained for 4 h, these solutions were defoamed in the vacuum drying oven for 30 min. Afterwards, the solutions were poured into petri dishes to cast into membranes, which were placed into the drying oven at 50 °C. After 3 days, transparent composite membranes were obtained.

### 2.3. Characterizations

Fourier transform infrared spectroscopy (FTIR): The FTIR results of the as-prepared samples were acquired with an NICOLETNEXUS470-type spectrometer provided by Perkin-Elmer Company, Waltham, MA, USA. The wavelengths were in the range of 4000–400 cm^−1^, and the resolution was 4 cm^−1^.

Field emission scanning electron microscopy (FESEM): The surface and cross-section morphologies of the composite membranes were obtained with an S-4800-type field emission scanning electron microscope (HITACHI Company, Tokyo, Japan). The acceleration voltage was 3 kV. The samples were coated with gold for 30 s for electric conduction.

Polarization microscopy (POM): The morphologies of the CNC suspension and composite membranes were obtained with a Nikon ECLPSE E200 polarization microscope (Nikon company, Tokyo, Japan).

Thermogravimetric analysis (TG): After being dried at 60 °C for 2 h, 5 mg of the samples was analyzed with a Q-500-type integrated thermo analyzer (American Security Products Company, Fontana, CA, USA) in N_2_ atmosphere. The heating rate was 10 °C/min, and the temperatures were in the range of 30–700 °C.

Tensile test: The membrane samples were cut into strips with a length of 70 mm, width of 10 mm, thickness of 0.1 mm, and gauge length of 50 mm. These strips were tested at room temperature with a UTM4503SLXY-type universal material testing machine made in China (SUNS company, Shenzhen, China). The tensile rate was 2 mm/min. According to the standard GB/T 1040.3-2006, the tensile strength (MPa) and elongation at break (%) were measured. Each membrane was tested five times.

X-ray diffraction (XRD): The composite membranes were detected with an X’Pert PRO X-ray diffractometer (PANalytical, Alemlo, Netherlands). 2θ of 5°–70° was scanned.

Zeta potential: a Zetasizer Nano ZS90 particle size & zeta potential analyzer (Malvern Instruments Ltd., Malvern, UK) was employed to measure the particle size and zeta potential. 0.5 wt % dispersion was tested at 25 °C and pH of 7.0. Each sample was tested three times.

Contact angle (CA): A sample was placed on the horizontal table of a JY-PHb-type contact angle analyzer (Jinhe Instruments Co., Ltd., Chengde, China). 2.5 μL of deionized water was laid on the surface of the sample, and the contact angle was measured three times with the aid of software.

Swelling test: a membrane sample (30 mm × 30 mm) was dried in an oven at 60 °C for 4 h. The initial mass was *Wi*. Then, the dried sample was immersed in deionized water at 25 °C for 2 min, 10 min, 30 min and 4 h. At each time, the sample was withdrawn and the free water over its surface was removed with filter paper. The hydrated mass was *Ws*. The swelling percentage was calculated as follows: swelling percentage (%) = $\left[\frac{Ws-Wi}{Wi}\right]\times 100$. Each membrane was tested three times.

## 3. Results and Discussion

### 3.1. Characterizations of CNC

**Figure 5:**
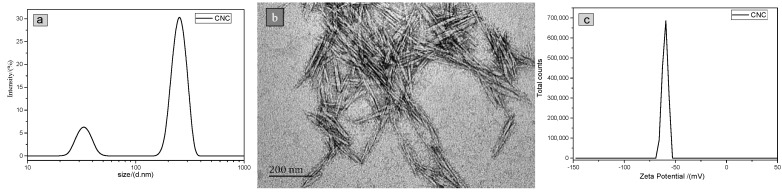
(**a**) Particle size distribution, (**b**) TEM image, and (**c**) Zeta potential distribution of CNC.

The particle size distribution result of CNC shown in Figure 5a exhibited two peaks, which result from the rod-like rather than spherical morphology of CNC. Hence, the smaller peak represents the diameter of the CNC with an average size of 16.2 nm, while the larger one represents the length of the CNC with an average size of 126.3 nm. These sizes are consistent with the TEM results (Figure 5b). The Zeta potential measured is shown in Figure 5c. The minus value indicates that the CNC prepared was an anionic polymer. If the Zeta potential of an aqueous dispersion is higher than +30 or lower than −30 mV, this dispersion should be well and stably dispersed (From page 243 of Zetasizer-Nano series user manual). The peak Zeta potential value was −59.37 mV, indicating that the CNC was well and stably dispersed.

### 3.2. Polarizing Microscopy Results of CNC/CS Composite Membranes

Because of the liquid crystal nature of CNC, it has birefringence performance, which makes CNC luminous in the view field of a polarizing microscope. In other words, the luminous region in the view field reflects the distribution situation of CNC.

**Figure 6:**
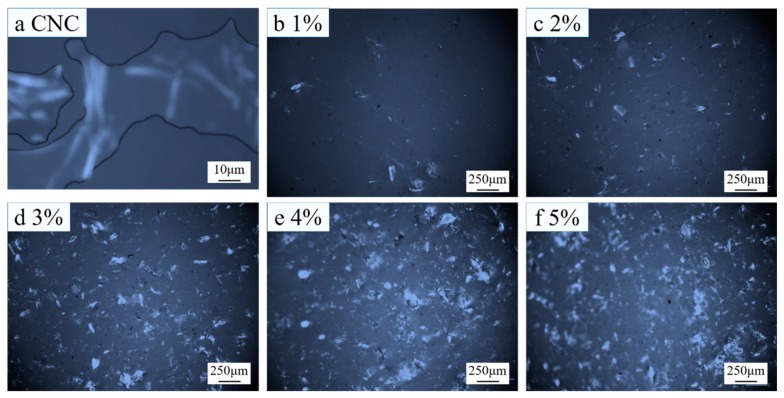
Polarizing microscopy results of (**a**) CNC suspension and (**b**–**f**) composite membranes prepared with 1–5% CNC suspensions (Method 1).

**Figure 7:**
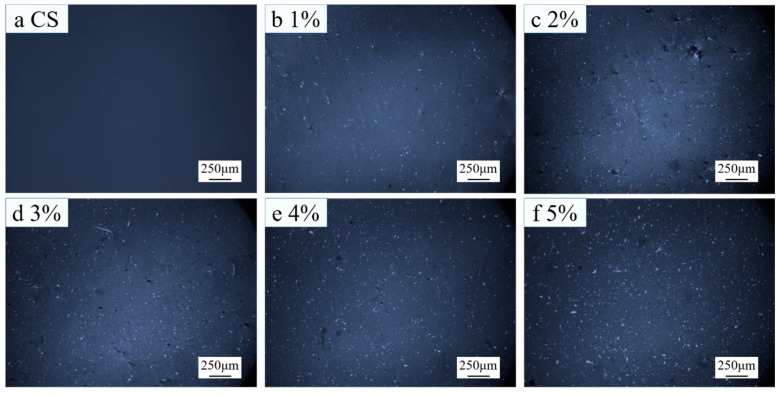
Polarizing microscopy results of (**a**) CS and (**b**–**f**) composite membranes prepared with 1–5% CNC suspensions (Method 2).

Figure 6a shows the polarizing microscopy result of the CNC suspension. Apparently, CNC was rod-like. Because chitosan has no liquid crystal property, chitosan was invisible, as shown in Figure 7a.

As shown in Figure 6b–f, CNC was lumpy in composite membranes. With the increase of CNC content, the lumps grew and the number of lumps increased. This indicates that the CNC agglomerated in composite membranes prepared with Method 1, and the agglomeration became more and more serious with the increase of CNC content.

As shown in Figure 7b–f, in the composite membranes prepared with Method 2, with the increase of CNC content, the number of luminous spots increased gradually, but the sizes of lumps were evidently smaller than those prepared with Method 1, indicating that the CNC was uniformly dispersed in the composite membranes prepared with Method 2.

### 3.3. SEM Images of CNC/CS Composite Membranes

**Figure 8:**
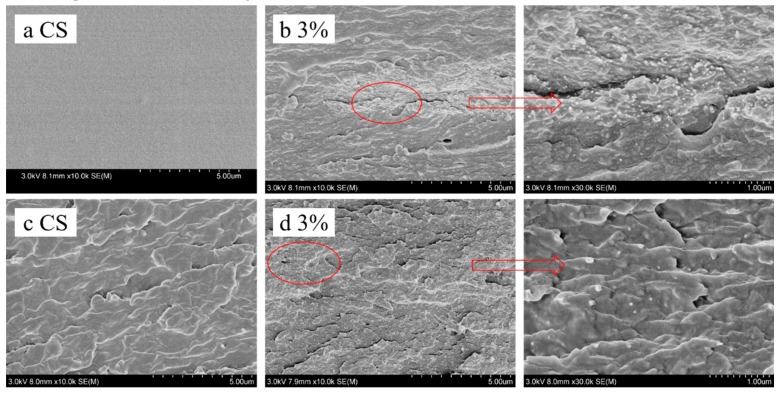
SEM images of (**a**) surface of CS membrane, (**b**) cross section of 3 wt % CNC composite membrane (Method 1), (**c**) cross section of CS membrane, and (**d**) cross section of 3 wt % CNC composite membrane (Method 2).

The presence of a large number of bubbles or aggregates of impurities in a composite membrane leads to stress concentration, which greatly reduces the mechanical properties and stability of this material. Figure 8a shows that the surface of pure-chitosan membrane was very smooth and dense, and bubbles were not observed. These phenomena indicate that the vacuum defoaming treatment was successful, ensuring good mechanical properties and stability of the composite membranes.

In the cross-section of the CNC/CS composite membrane (Figure 8b) prepared with Method 1, many small spherical particles were observed, but in the cross-section of the CS membrane (Figure 8c), no spherical particles could be found. It is implied that these small particles are CNC. The enlargement of Figure 8d shows that these particles had diameters of about several nanometers to tens of nanometers. In Figure 8b, many CNC were concentrated in the middle, while the CNC were rare on the upper and lower sides, showing that CNC was not uniformly dispersed in the composite membranes prepared with Method 1. In contrast, the dispersion of CNC on the cross section of CNC/CS composite membranes prepared with Method 2 (Figure 8d) was more uniform, which shows that the dispersion of CNC was good in these membranes.

### 3.4. Mechanical Properties of CNC/CS Composite Membranes

The Table 1 indicates tensile strength (TS) and elongation at break (EB) values with standard deviations, respectively.

**Figure 9:**
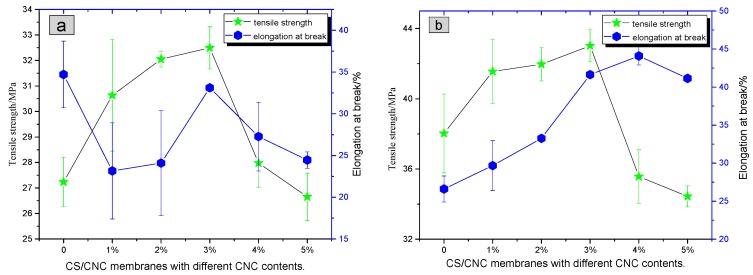
Effect of CNC contents on the mechanical properties of CNC/CS composite membranes prepared with (**a**) Method 1 and (**b**) Method 2, respectively.

(1) The effects of different dispersion methods on the properties of composite membranes: when the CNC content was 3%, the tensile strength of membrane (43.0 MPa) prepared with Method 2 was 32.3% higher than that (32.5 MPa) prepared with Method 1. On the other hand, the elongation at break (41.6%) was improved by 25.7% compared to that (33.1%) prepared with Method 1. (The tensile strength of the chitosan composite film prepared by the Method 1 is similar to that prepared by Huang et al. [29]). These results indicate that the mechanical properties of the membrane prepared with Method 2 were better. In addition, the fluctuation of standard deviations shown in Figure 9b is smaller than that shown in Figure 9a, indicating that the tensile stability of the composite membrane prepared with Method 2 was higher than that prepared with Method 1.

CNC was the dispersion phase and CS was the continuous phase in the composite membranes. In Method 2, CNC was well dispersed in CS according to the results of SEM and POM. Therefore, phase separation in relative membranes was not easy, and stress concentration was reduced. Additionally, the interactions between CNC and CS molecules, such as electrostatic association and hydrogen bonding, effectively improved the mechanical properties and stability of the relative composite membranes.

(2) The effect of CNC content on the mechanical properties of composite membranes: Figure 9b shows that the tensile strength of composite membranes prepared with Method 2 exhibited a volcanic trend against the CNC content. When the mass fraction of CNC was 3%, the tensile strength reached a maximum of 43.0 MPa, which was 13.2% higher than that of pure CS membrane (38.0 MPa). The elongation at break also exhibited a volcanic trend against the CNC content. When CNC content was 4%, a maximum of 44.1% was reached. The maximum value was 65.8% higher than that of pure CS membrane (26.6%). The composite membrane with the CNC content of 3% showed the best mechanical performance. Compared with the CS membrane, the tensile strength and elongation at break were increased by 13.2% and 56.4%, respectively.

CNC had a large length/diameter ratio and excellent mechanical properties. The interactions between CNC and CS molecules, such as electrostatic association and hydrogen bonding, produced an interactive network, which improved the overall mechanical properties of composite membranes. The dispersion of 3 wt % CNC was better than that of other contents, so the mechanical properties of the relative membrane were the best.

### 3.5. FTIR Spectra of CNC/CS Composite Membranes

Because the composite membranes prepared with Method 2 showed better performances, the composite membranes of the following studies were prepared by Method 2

**Figure 10:**
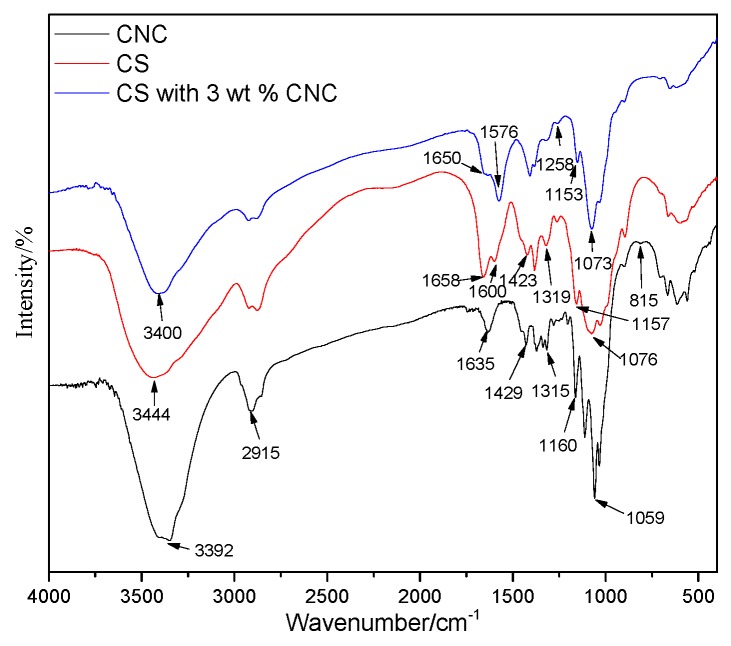
FTIR spectra of CNC, CS and CNC/CS composite membranes.

The molecular skeletons of CS and CNC membranes are similar, so they have many of the same absorption peaks. As shown in Figure 10, the broad band around 3400 cm^−1^ corresponds to the stretching vibration of O–H and N–H. The band at 2915 cm^−1^ corresponds to the stretching vibration of C–H. The band at 1429 cm^−1^ corresponds to the symmetrical bending vibration of C–H_2_. The band at 1315 cm^−1^ corresponds to the swinging symmetrical bending vibration of C–H_2_. The band at 1160 cm^−1^ corresponds to the asymmetric stretching vibration of C–O–C glycosidic bonds. The band at 1059 cm^−1^ corresponds to the stretching vibration of –C–O [46].

In the FTIR spectra of CNC membranes, the band at 1635 cm^−1^ corresponds to the stretching vibration of glucose lactone. The bands at 1250 and 815 cm^−1^ correspond to the asymmetric vibration of S=O and symmetric vibration of C–O–S, respectively. The presence of these characteristic bands indicates the introduction of sulfate monoester groups [47] in the course of cellulose acidolysis.

In the FTIR spectra of CS membranes, the band at 1658 cm^−1^ corresponds to the stretching vibration of C=O in amides, and the band at 1600 cm^−1^ corresponds to the bending vibration of –NH [48].

In the FTIR spectra of CNC/CS composite membranes, the band at 1258 cm^−1^ corresponds to the asymmetric vibration of S=O in CNC, indicating that CNC had been successfully introduced into the composite membranes. The band due to the stretching vibration of O–H and N–H was shifted from 3444 to 3400 cm^−1^. Generally, the formation of hydrogen bonds leads to the uniformity of electron cloud density, and the stretching vibration frequency will be lowered. Similarly, in this study, the FTIR results implied that strong hydrogen bonds were formed between CS and CNC molecules. Accordingly, the band corresponding to the stretching vibration of C=O in amides in CS was shifted from 1658 to 1650 cm^−1^, and the band corresponding to the bending vibration of –NH was shifted from 1600 to 1576 cm^−1^, with the signal intensity obviously increased. These results indicate that the –NH_2_ and C=O groups on CS intensely interacted with the –OH groups on CNC, which enhanced the bonding at the interface and effectively improved the mechanical performance of CS [49].

### 3.6. XRD Patterns of CNC/CS Composite Membranes

**Figure 11:**
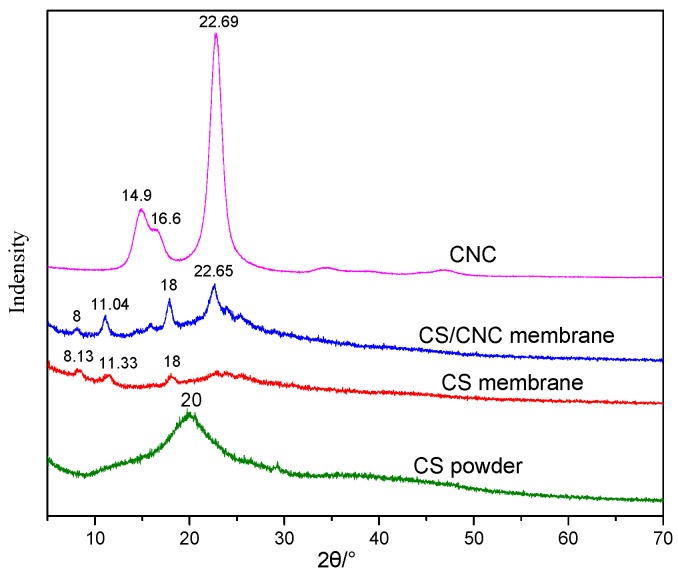
XRD patterns of CNC, CNC/CS membrane, CS membrane, and CS powder.

As illustrated in Figure 11, in the XRD pattern of CNC, obvious diffraction signals at about 14.9° (1$\bar{1}$0), 16.6° (110), and 22.69° (200) were observed, i.e., cellulose I crystal form [50,51], which consists of parallel molecular chains and contains a large number of hydroxyl groups enclosed in the crystal cells. These hydroxyl groups were closely connected with a great number of hydrogen bonds. A broad diffraction peak at 20° was observed in the XRD pattern of chitosan powder, but different diffraction peaks at 8.13°, 11.33° and 18° were observed in the pattern of chitosan membrane. These results indicate that the original crystal form of chitosan was destroyed after the solution of chitosan in acetic acid. Likewise, the XRD pattern of CNC/CS composite membrane also showed similar diffraction peaks at 8.13°, 11.33° and 18°. In addition, a new signal corresponding to CNC was observed at about 22.65°, indicating the successful combination of CNC and CS. The crystallinity of CNC/CS composite membranes was higher than that of CS membranes, which implies that the strong interactions between CS and CNC made the crystalline structure of the composite membrane more ordered. That is the reason why the mechanical properties of CNC/CS membranes were improved.

### 3.7. Thermal Analysis

**Figure 12:**
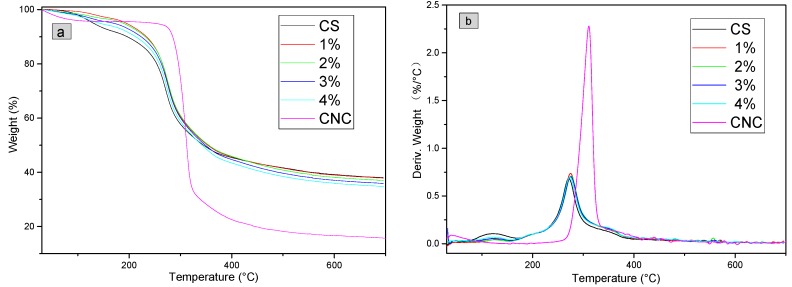
(**a**) TG and (**b**) DTG analysis results of CNC/CS composite membranes.

As shown in Figure 12a, at below 100 °C, the weight loss in the TG profile of CNC was attributed to the volatilization of a small amount of water. The DTG profile of CNC (Figure 12b) showed an obvious signal at 250–350 °C, and the weight loss was as high as 66.7%. This signal represents the pyrolysis of CNC. At above 500 °C, the weight loss was negligible, and the percentage of carbon residue was 15.7%.

As shown in Figure 12b, the weight loss rate of CS has two distinct peaks, the first peak range was 100~160°C (mass loss was 5.5%) and the second peak range was 200–400 °C (mass loss was 44.2%), which was the main cracking stage of CS thermal decomposition, mainly due to the breakage of the glycosidic bonds between glucosamine and N-acetylglucosamine rings [52,53]. At above 500 °C, the weight loss was negligible, and the percentage of carbon residue was 37.9%.

The DTG profiles of composite membranes and CS (Figure 12b) are similar, especially the signal at ~280 °C. The intensities of signal at ~120 °C of the composite membranes were smaller than that of the CS membrane, which indicates that the weight loss of the composite membrane was slower. The TG data in Figure 12a proved that in the range of 120–320 °C, the weights of CNC/CS composite membranes were greater than that of the CS membrane, indicating that the composite membranes had a better heat resistance performance compared to the pure CS membrane at below 320 °C. At above 400 °C, carbonization took place, and the percentages of carbon residues of these composite membranes were between those of the CS and CNC membranes. With the increase of CNC content, the percentage of carbon residue was reduced gradually.

The main positive factors affecting the thermal stability of polymers include the rigidity, crystallinity and intermolecular interactions of polymer chains [52]. The addition of CNC improved the overall rigidity and crystallinity of composite membranes. The components in CNC/CS composite membranes were stably bonded by electrostatic association and hydrogen bonds rather than simple physical interactions. The electrostatic association and hydrogen bonds rendered the breakage, dehydration, decarboxylation and decarbonylation of glycosidic bonds, C–H bonds, C–O bonds and C–C bonds in cellulose and chitosan molecules more difficult. Therefore, the addition of CNC improved the heat resistance of CNC/CS membranes.

### 3.8. Contact Angle of CNC/CS Composite Membranes

**Figure 13:**
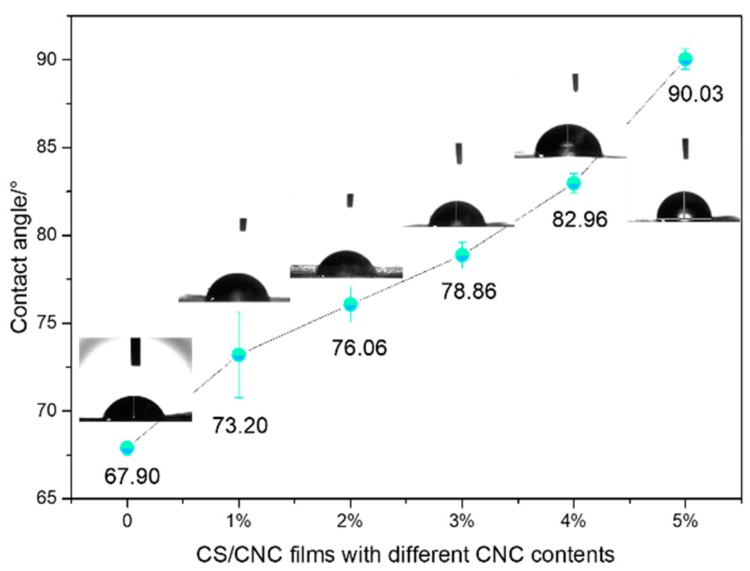
Effect of CNC content on the contact angle properties of CNC/CS composite membranes.

As illustrated in Figure 13, the contact angle of the pure CS membrane was 67.9°. With the increase of CNC content, the contact angle became larger and larger. When the CNC content reached 5 wt %, the contact angle was up to 90.03°, showing that CNC improved the hydrophobic performance of composite membranes, which is beneficial for the water resistance of chitosan membrane. The contact angle of pure cellulose membrane was 70° [54], and those of pure CNC and CS membranes are smaller than 70°. In contrast, those of the composite membranes are greater than 70°, implying that CNC and CS molecules were closely bonded by electrostatic association, hydrogen bonds and so on, which improved the hydrophobic performance of CNC/CS membranes.

### 3.9. Swelling Properties of CNC/CS Composite Membranes

**Figure 14:**
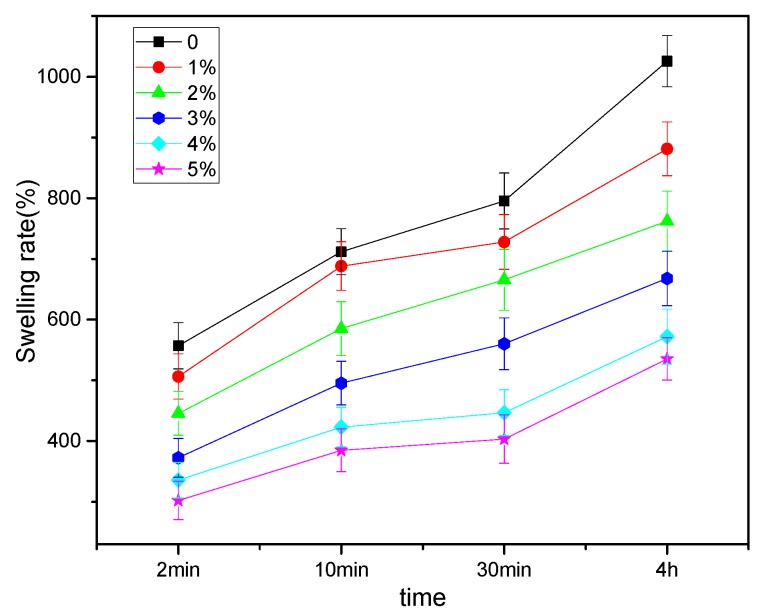
Effect of CNC content on the swelling properties of CNC/CS composite membranes.

As shown in Figure 14, the swelling percentages of the composite membranes increased over time. On the other hand, at a certain immersion time, the percentages decreased with the increase of CNC content. For instance, when the immersion time was 4 h, the swelling percentages of pure CS and 5 wt % CNC composite membranes were 1025.8% and 535.3%, respectively. The latter was about half of that of the former, demonstrating that the presence of CNC could effectively weaken the swelling performance of CNC/CS membranes. The reasons are the following: first, the crystallinity of CNC was high. Secondly, the strong electrostatic association and hydrogen bonding between CNC and CS molecules in the composite membranes enhanced the network structure, limited the flexibility of CS molecular chains, and hindered the permeation paths of water molecules, demonstrating that CNC could improve the water resistance of CNC/CS membranes.

### 3.10. Biodegradation of CNC/CS Composite Membranes

**Figure 15:**
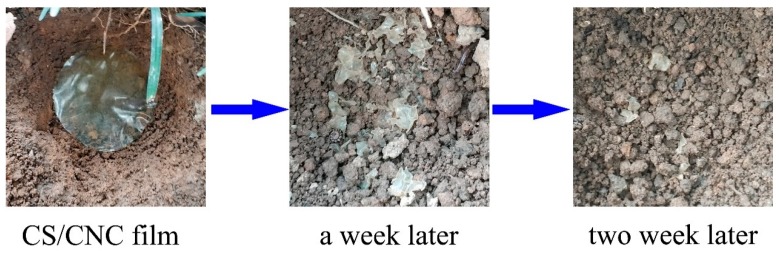
Biodegradation performances of the composite membrane.

A 3 wt % CNC composite membrane was buried in soil for the study on its biodegradation performance. As shown in Figure 15, a week later, the membrane was decomposed into pieces. Two weeks later, only tiny debris remained, and the bulk had been decomposed by microorganisms, indicating that this composite membrane had good biodegradability.

## 4. Conclusions

CNC/CS composite membranes were successfully prepared via mechanical mixing and solution casting. The membranes were compact, uniform and bubble-free. The morphologies of the surface and cross-section were observed by SEM and POM. The results showed that the composite membranes prepared with Method 2 had better dispersion performances, but the dispersion performances became poorer if the CNC concentration was too high. The dispersion performance was relatively good when the CNC content was 3%, when the tensile strength and elongation at break of the composite membrane were increased by 13.2% and 56.4%, respectively, compared to the pure CS membrane. These results showed that the interfacial compatibility between CNC (dispersion phase) and CS (continuous phase) was quite good. The interactions including electrostatic association and hydrogen bonds between CNC with large length-diameter ratios and CS molecules caused the formation of an interactive network structure, which improved the mechanical properties, heat resistance and water resistance of the composite membrane. This type of safe, non-toxic, renewable and biodegradable CNC/CS composite membranes, as a novel food packaging material, may replace petroleum-based polymers for the carbon emissions reduction.

## Figures and Tables

**Table 1:** Effects of CNC contents on the mechanical properties of CNC/CS composite membranes.

CNC%	TS/MPa		EB/%	
	Method 1	Method 2	Method 1	Method 2
0	27.2 ± 1.0	38.0 ± 2.3	34.7 ± 4.0	26.6 ± 1.7
1	30.6 ± 2.2	41.6 ± 1.8	23.2 ± 5.8	29.7 ± 3.3
2	32.1 ± 0.3	42.0 ± 0.9	24.1 ± 6.3	33.3 ± 0.2
3	32.5 ± 0.8	43.0 ± 0.9	33.1 ± 0.3	41.6 ± 0.3
4	28.0 ± 1.0	35.6 ± 1.5	27.3 ± 4.1	44.1 ± 1.2
5	26.7 ± 0.9	34.4 ± 0.6	24.5 ± 1.0	41.1 ± 0.1
